# Validation and psychometric evaluation of the Short Warwick-Edinburgh Mental Well-Being Scale (SWEMWBS) among Czech adolescents using Item Response Theory

**DOI:** 10.1186/s12955-024-02280-9

**Published:** 2024-08-19

**Authors:** Radka Hanzlová, Aleš Kudrnáč

**Affiliations:** Institute of Sociology of the Czech Academy of Sciences, Prague, Czech Republic

**Keywords:** SWEMWBS, IRT, Validation, Psychometric analysis, Mental well-being, Czechia, Adolescents, CZEPS

## Abstract

**Background:**

The topic of adolescent mental health is currently a subject of much debate due to the increasing prevalence of mental health problems among this age group. Therefore, it is crucial to have high-quality and validated mental well-being measurement tools. While such tools do exist, they are often not tailored specifically to adolescents and are not available in Czech language. The aim of this study is to validate and test the Czech version of the Short Warwick-Edinburgh Mental Well-Being Scale (SWEMWBS) on a large sample of Czech adolescents aged 15 to 18 years.

**Methods:**

The analysis is based on data from the first wave of the Czech Education Panel Survey (CZEPS) and was mainly conducted using Item Response Theory (IRT), which is the most appropriate method for this type of analysis. Specifically, the Graded Response Model (GRM) was applied to the data. This comprehensive validation study also included reliability and three types of validity (construct, convergent and criterion) testing.

**Results:**

The study found that the Czech version of the SWEMWBS for adolescents aged 15 to 18 years (*N* = 22,498) has good quality and psychometric properties. The data was analysed using the GRM model as it met the assumptions for the use of IRT. The estimated parameter values by GRM demonstrated good discriminant and informative power for all items, except for item 7, which showed poorer results compared to the others. However, excluding it from the scale would not enhance the overall quality of the scale. The five-category response scale functions effectively. Additionally, the results demonstrated high reliability, and all types of validity tested were also confirmed.

**Conclusions:**

The Czech version of the SWEMWBS for adolescents has been validated as a psychometrically sound, reliable and valid instrument for measuring mental well-being. It can therefore be used with confidence in future studies.

**Supplementary Information:**

The online version contains supplementary material available at 10.1186/s12955-024-02280-9.

## Background

Recent years have witnessed a growing debate about adolescents’ mental well-being. Mental well-being is the positive aspect of mental health and not merely the absence of disease that fluctuates over time in response to both internal and external factors [1]. The World Health Organisation (WHO) defines mental health as “a state of well-being in which individual realizes his or her own abilities, can cope with the normal stresses of life, can work productively and is able to make a contribution to his or her community” [2]. This topic has generated a wide interest as a result of the increasing number of adolescents reporting poor mental health. According to the most recent estimation from Global Burden of Disease Study, around 19% of adolescents aged 15–19 living in the EU have mental health conditions [3]. However, various studies suggest that mental health problems among adolescents rapidly increased after 2020 in response to the COVID-19 pandemic [4]. Although Generation Z, i.e. people born between the mid-1990s to 2010, is often described as a “snowflake generation”, young people face unprecedented challenges such as excessive social media use and the consequences of the COVID-19 pandemic, which increased levels of anxiety, depression and feelings of isolation [5]. Adolescents are thus widely considered to be a vulnerable population susceptible to mental health issues [6].

Adolescence is not only an important phase of life during which individuals undergo major biological and physiological changes, but also a developmentally sensitive time for an individual’s mental health. Indeed, the majority of mental health disorders in adults typically begin during adolescence [7]. Consequently, having a validated tool for measuring mental well-being among adolescents is crucial for following trends in mental well-being and identifying potential means of support or intervention [8]. Although there are several validated tools for measuring mental well-being, they are mostly developed for the adult population and are scarcely available in the Czech language. Therefore, the aim of this article is to validate a mental well-being measure for Czech adolescents.

According to recent data from a large survey conducted among Czech early adolescents using validated tools for measuring the mental diseases of children and adolescents (e.g., WHO-5, PHQ-9, GAD-7) [9], over 50% exhibited signs of impaired well-being, 40% showed signs of moderate to severe depression, and 30% exhibited signs of anxiety [10]. However, we need a properly validated measure to assess overall mental well-being in the Czech language.

Using data from the Czech Education Panel Survey [11], we validate and test the psychometric properties of the Short Warwick-Edinburgh Mental Well-Being Scale (SWEMWBS) on a representative sample of 22,498 Czech adolescents (aged 15 to 18 years) through Item Response Theory (IRT). While validated studies of this scale exist in English [e.g., 12, 13] as well as in some other languages [e.g., 14], to date only one study has specifically focused on adolescents and utilized IRT [15], which is considered the best method for testing the psychometric properties and quality of measurement tools. The primary objective of this study is to test and validate SWEMWBS utilizing IRT for Czech adolescent population. Furthermore, this study will be the first of its kind to be conducted in the Central European region.

## Methods

### Sample and data

The Czech-language adaptation of the SWEMWBS for adolescents was based on data collected through an online questionnaire (CASI method) in October and November 2023 within the CZEPS project (https://czeps.soc.cas.cz/en) [11]. Respondents were first-year secondary school students aged 15 to 18. Only respondents who answered all SWEMWBS items were included in the analysis. The final research sample (*N*) comprised 22,498 students aged 15 to 18 (mean age 15.6 years), of which 10,757 were male (47.8%), 11,045 female (49.1%) and 696 other (3.1%). For further details regarding the structure of the research sample, see Table A1 in the Appendix.

### Measures

#### The Short Warwick-Edinburgh Mental Well-Being Scale (SWEMWBS)^1^

The SWEMWBS is a shortened version of the original 14-item WEMWBS by Stewart-Brown et al. [12] The SWEMWBS consists of seven positively-worded items covering both aspects of mental well-being – feeling good and functioning well – that respondents rated using a 5-point Likert scale (1 ‘*none of the time*’, 2 ‘*rarely*’, 3 ‘*some of the time*’, 4 ‘*often*’, 5 ‘*all of the time*’). The evaluation is calculated by summing the scores of each item. The total raw score ranges from 7 to 35 (a higher value means a higher level of mental well-being). However, for analyses, it is necessary to transform the raw score into a metric score [12]. The SWEMWBS has been translated into Czech through the TRAPD approach, which is an acronym for the steps of the translation process; specifically Translation, Review, Adjudication, Pretest, and Documentation [16]. Three independent experts translated the items into Czech, followed by a review and assessment, and the most appropriate wording was selected. Then, pilot testing of the Czech translation was conducted among 74 students from two secondary schools via an online questionnaire. The results of the pilot testing indicated that only minor changes were necessary, such as alterations to the word order and the use of synonyms. All stages of the translation process were documented. The original wording and the final Czech translation are shown in Table 1.

**Table 1:** ** Final** Czech translation of the SWEMWBS

No	Original English-language version	Czech-language translation
1	I’ve been feeling optimistic about the future.	Svou budoucnost jsem viděl/a optimisticky.
2	I’ve been feeling useful.	Připadal/a jsem si užitečný/á.
3	I’ve been feeling relaxed.	Cítil/a jsem se uvolněně.
4	I’ve been dealing with problems well.	S problémy jsem se vyrovnával/a dobře.
5	I’ve been thinking clearly.	Byl/a jsem schopen/schopna jasně přemýšlet.
6	I’ve been feeling close to other people.	Cítil/a jsem spřízněnost s ostatními lidmi.
7	I’ve been able to make up my own mind about things.	Dokázal/a jsem si na věci udělat vlastní názor.

*Instruction*: “Choose how often you had the following thoughts or feelings in the last two weeks. / Vyberte, jak často jste v posledních dvou týdnech měl/a následující myšlenky nebo pocity.

*Response scale*: 1 = none of the time/nikdy, 2 = rarely/zřídka, 3 = some of the time/občas, 4 = often/často, 5 = all of the time/vždy

For assessment of the criterion-related validity of the Czech version of the SWEMWBS, the relations with other similar instruments were investigated. The construct used and how they were assessed are described below.

#### Overall life satisfaction

Life satisfaction was measured by a traditional one-item question: “*All things considered, how satisfied are you with your life as a whole nowadays? Please answer on a scale of 0 to 10, where 0 means ‘extremely dissatisfied’ and 10 means ‘extremely satisfied’.”*

#### Happiness

Positive affect was measured by a simple question on happiness asking respondents: “*Taking all things together, how happy would you say you are?*” They answered on an 11-point response scale from 0 ‘*extremely unhappy*’ to 10 ‘*extremely happy*’.

#### General health

One question was used to assess subjective health with the following wording: “*Would you say that your health in general is…* (1) *poor*, (2) *average*, (3) *good*, (4) *very good*, or (5) *excellent*?”.

#### General Anxiety Disorder-2 (GAD-2)

The GAD-2 is an ultra-short version of the original 7-item version containing two items (*Feeling nervous, anxious, or on edge*; *Not being able to stop or control worrying*) measuring anxiety [17]. Respondents were asked, “*How often have they been bothered by these problems over the last two weeks?*” and answered on a 4-point response scale (0 ‘*Not at all*’, 1 ‘*Several days*’, 2 ‘*More than half days*’, 3 ‘*Almost every day*’).

#### Patient Health Questionnaire-2 (PHQ-2)

Depression was measured by the 2-item PHQ-2 [18], which contains nine items in the original version. The wording of the question and response scale is the same as for GAD-2. The wording of the items is as follows: *Little interest or pleasure in doing things* and *Feeling down, depressed or hopeless*.

#### Brief Resilience Scale (BRS)

The BRS is a short 6-item scale created to assess the perceived ability to bounce back or recover from stress [19]. The scale includes three positively (items 1, 3, and 5), and three negatively (items 2, 4, and 6) worded items rate on a 5-point response scale (1 ‘*strongly disagree*’, 2 ‘*disagree*’, 3 ‘*neither agree nor disagree*’, 4 ‘*agree*’, 5 ‘*strongly agree*’). The total score is calculated as the sum of the individual items after recoding.

### Statistical analysis plan and methods

The validation process was conducted in several steps using various methods. First, descriptive statistics were performed. Subsequently, the assumptions for using IRT, the main method for testing the psychometric properties of the Czech translation of the SWEMWBS, were tested. The scale items have five ordered categories and are polytomous, so we applied two models to them and compared their results, namely the General Partial Credit Model (GPCM) [20] and the Graded Response Model (GRM) [21]. Using the more appropriate model, we estimated one discriminant parameter (*a*) and four threshold parameters (*b*) for each item (the number of threshold parameters is always one less than the number of items on the response scale). The discriminant parameter indicates the item's relationship to the scale and its ability to differentiate between respondents with different levels of the measured concept, also known as the latent trait (theta, *θ*). The parameter *a* typically ranges from 0 to 2, but theoretically, it can range from –∞ to + ∞ [22]. The interpretation of parameter *b*, which typically ranges from –3 to + 3 [23], varies depending on the model used. For the GPCM, it represents the value of the latent variable required to move between two adjacent categories on the response scale, while for the GRM, it denotes the 50% probability that the respondent will select the given category or a higher category on the response scale. The evaluation was also conducted using illustrative graphs such as item characteristic curve (ICC), category characteristic curve (CCC), item information function (IIF), and test information function (TIF). The final step involved testing reliability by using the coefficients Cronbach's alpha and McDonald's omega, and various types of validity, namely construct validity through confirmatory factor analysis (CFA), convergent validity based on Average Variance Extracted (AVE) and Composite Reliability (CR), and criterion-related validity by correlation with other relevant measures.

Data preparation and basic analyses, including descriptive statistics, reliability, validity, and unidimensionality testing, were conducted using SPSS 27, CFA tested construct validity in Mplus 7.4, and IRT analysis was performed in STATA 17 and R using the *mirt* package.

## Results

### Descriptives

Table 2 shows the descriptive statistics and the results of testing normality for the total scale and each SWEMWBS item. The mean scores for all items were above average and ranged from 3.01 (item 2) to 4.19 (item 7). The overall mean of the scale was 21.44. The corrected-item total correlation for most items was greater than 0.5, indicating a strong relation to the scale; however, where the value is lower, mainly for item 7 (0.39), it remains above the acceptability threshold [24]. All items can be considered normally distributed because the limit value for both skewness ( ≤|2|) and kurtosis ( ≤|7|) was not exceeded [25].

**Table 2:** Descriptive statistics for SWEMWBS 7 items (*N* = 22,498)

	***M***	***SD***	**Skewness**	**Kurtosis**	***r***_**it**_
I’ve been					
Item 1: feeling optimistic about the future	3.13	1.05	–⁠0.18	–⁠0.42	0.55
Item 2: feeling useful	3.01	1.04	–⁠0.09	–⁠0.47	0.65
Item 3: feeling relaxed	3.14	1.06	–⁠0.13	–⁠0.64	0.61
Item 4: dealing with problems well	3.16	1.17	–⁠0.14	–⁠0.84	0.62
Item 5: thinking clearly	3.37	1.04	–⁠0.28	–⁠0.46	0.62
Item 6: feeling close to other people	3.28	1.04	–⁠0.28	–⁠0.43	0.49
Item 7: able to make up my own mind about things	4.19	0.93	–⁠1.13	0.93	0.39
SWEMWBS	21.44^a^	4.21	0.52	1.31	

*M* mean, *SD* standard deviation, *r*_it_ corrected-item total correlation

^a^Mean on a range 7–⁠35

### Assumptions of IRT

The application of IRT requires the satisfaction of three assumptions: unidimensionality, local independence, and monotonicity. Unidimensionality was tested using the principal components analysis (PCA), which resulted in a clear extraction of one factor (with an eigenvalue of 3.39), explaining 48.41% of the variance (see Table A2 and Figure A1 in the Appendix). The local independence was tested using the Yen Q3 test [26], which measures the residual correlation between pairs of items. To ensure satisfactory results, the correlation should not exceed 0.20 [27]. The correlation between several items was slightly higher (at most 0.28) (see Table A3 in the Appendix). However, this assumption can still be considered fulfilled because the CZEPS survey and SWEMWBS fulfilled the main recommendation for maintaining local independence, which is thorough questionnaire preparation and positive item wording. The positive wording also corresponds to the type of response scale, with a higher score indicating a higher level of mental well-being. This is necessary to meet the last assumption of monotonicity. All three assumptions have been tested and met; therefore, IRT can be applied to the data.

### IRT analysis

The comparison of the GPCM and GRM models based on the log-likelihood, the Bayesian Information Criterion (BIC) and the Akaike Information Criterion (AIC) values indicated that the GRM model had a better fit (higher log-likelihood and lower AIC and BIC) (for more detail, see Table A4 in the Appendix). Therefore, the GRM model was used in the IRT analyses.

**Fig. 1:**
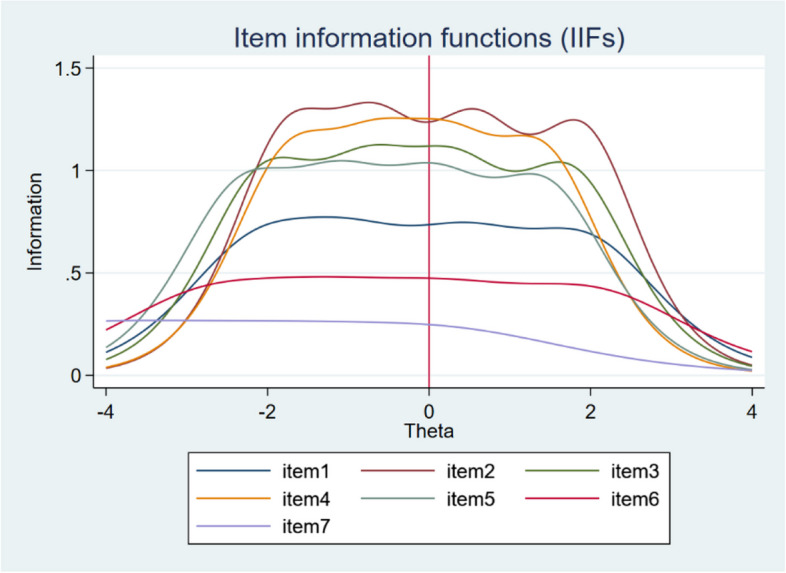
Item information functions (IIFs) for each item of the SWEMWBS with a vertical line at *θ* = 0

The discrimination parameter (*a*) and threshold parameter (*b*) values from GRM are shown in Table 3. The values of the discrimination parameter (*a*) range from 0.93 (item 7) to 2.18 (item 2), indicating good discriminant power. According to Baker [23], four items (2, 3, 4, and 5) can be described as ‘very highly’ discriminative, item 1 as ‘highly’ discriminative and items 6 and 7 as ‘moderately’ discriminative. These results are very clearly consistent with Fig. 1 of the Information item functions (IIFs), in which the items are ranked according to the information power corresponding to the value of the discrimination parameter (*a*). The least informative and least discriminating item (item 7) is ranked the lowest while conversely, the most discriminating and informative items (2 and 4) are ranked the highest and show variability in curve shape (as opposed to flatness, especially for item 7).

**Table 3:** Discrimination and thresholds parameters for SWEMWBS

	Discrimination parameter	Difficulty parameters for each threshold			
	*a*	*b1*	*b2*	*b3*	*b4*
Item 1	1.62	–2.11	–0.98	0.46	1.94
Item 2	2.18	–1.75	–0.66	0.57	1.91
Item 3	1.98	–2.04	–0.76	0.33	1.75
Item 4	2.06	–1.73	–0.66	0.26	1.43
Item 5	1.91	–2.32	–1.11	0.08	1.45
Item 6	1.27	–2.72	–1.27	0.23	2.05
Item 7	0.93	–4.93	–3.38	–1.65	0.21

Threshold parameters (*b*) were estimated for a total of 28 (four for each item). Their values range from –4.93 (item 7) to 2.05 (item 6). Item 7, in particular, exceeds the value of ± 3 and shows poorer functioning than the others (i.e., it captures significantly more respondents with lower mental well-being scores, as three out of the four threshold parameters are negative). The values for the other items are balanced in terms of positive and negative values, indicating that they measure and discriminate well among respondents along the latent trait continuum (mental well-being).

**Fig. 2:**
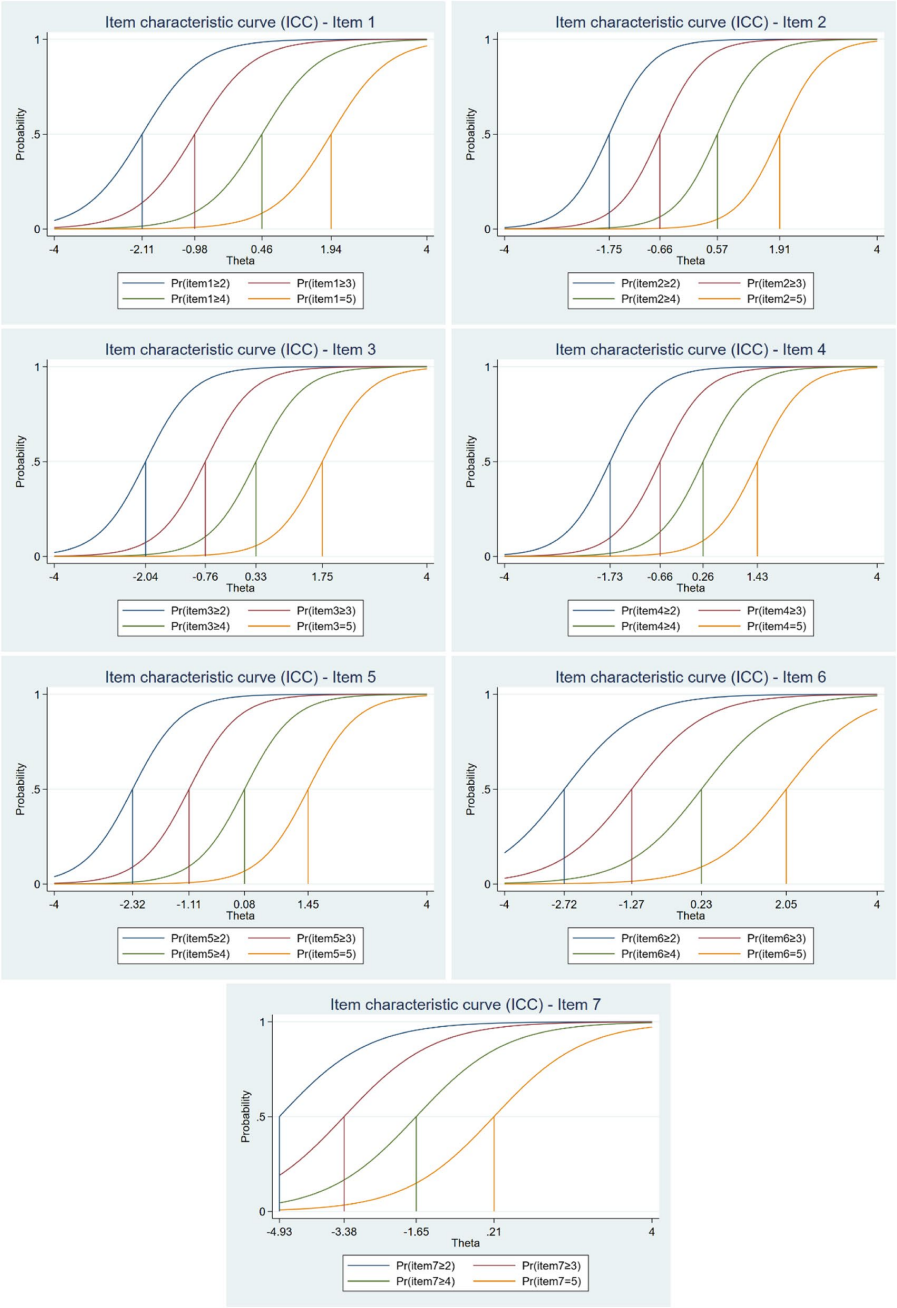
Item characteristic curves (ICCs) for each item of the SWEMWBS

The *b*-parameters reveal differences in the response scale categories between the items. This is particularly noticeable for item 7, where a lower latent trait level is required to select a higher category on the response scale. For example, to select category 5 on the response scale, the respondent must achieve a value of 0.21 for item 7, whereas for item 6, the value is 2.05. However, the study revealed that the response scale functioned effectively for all items, as the differences between categories were consistently minimal (for more detail, see Table A5 in the Appendix). These findings are further supported by the ICCs, as shown in Fig. 2.

**Fig. 3:**
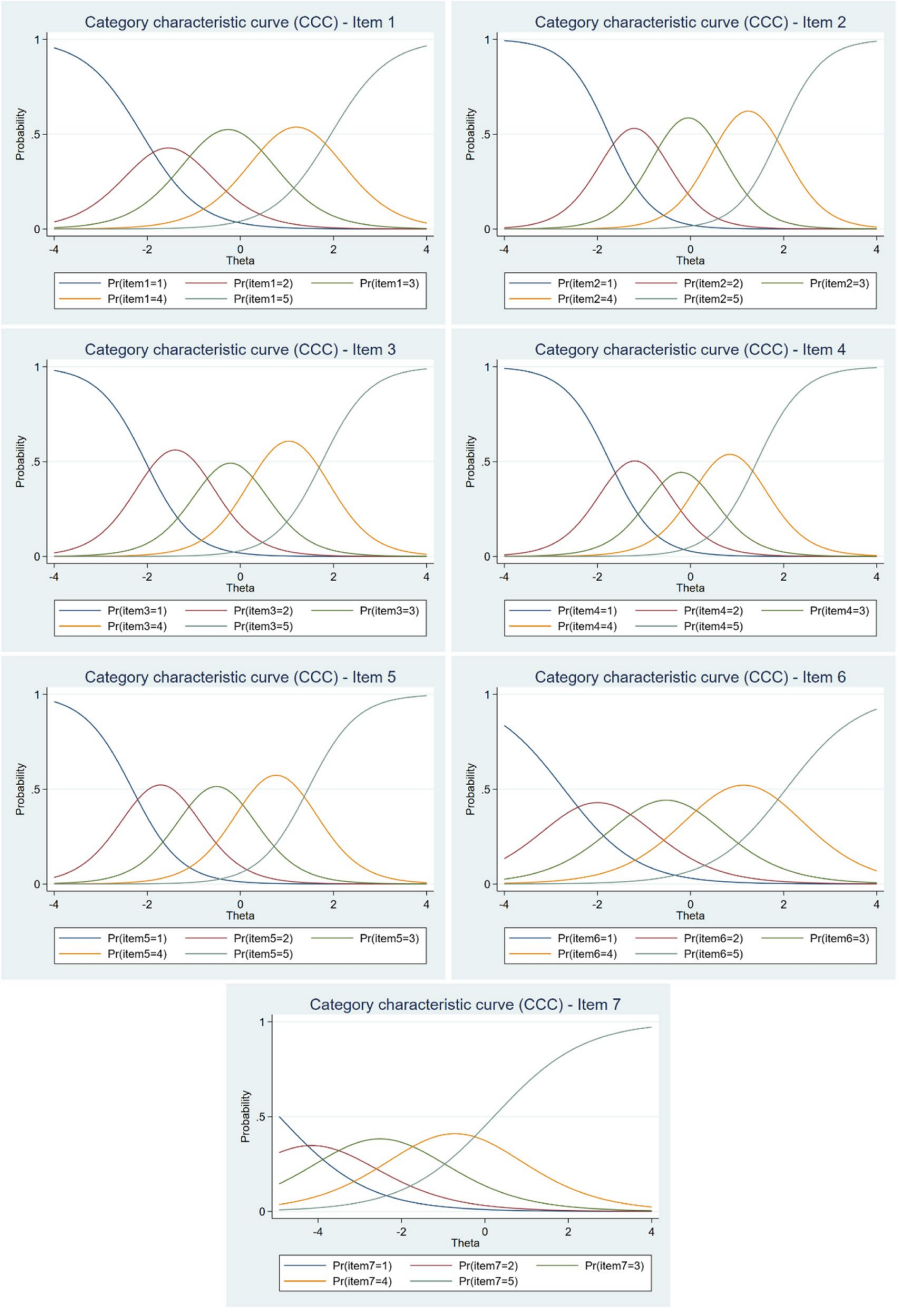
Category characteristic curves (CCCs) for each item of the SWEMWBS

The response scale’s functioning can also be analysed through the CCC. Figure 3 displays the CCC for each item from the SWEMWBS. The correct functioning of the response scale is characterised by the fact that each category is the most likely choice in some part of the latent trait continuum. This means that it has a clear peak and is not superimposed by the curve of another response category. Related to this is the assumption of monotonicity that the higher category on the response scale should be selected by the respondent with a higher latent trait level [28]. The shapes of the curves are influenced by parameter values, with a higher value of the parameter *a* (slope) resulting in a sharper peak and a more rapid change in the probability of selection between adjacent categories [29]. The results showed that the response scale works well for all items and has an appropriate number of response categories. However, it is also evident that the functioning of item 7 is not ideal. The response category curves are relatively flat and lack sharp peaks. This confirms the previously described results that this item is ineffective in distinguishing between respondents with positive latent trait values.

**Fig. 4:**
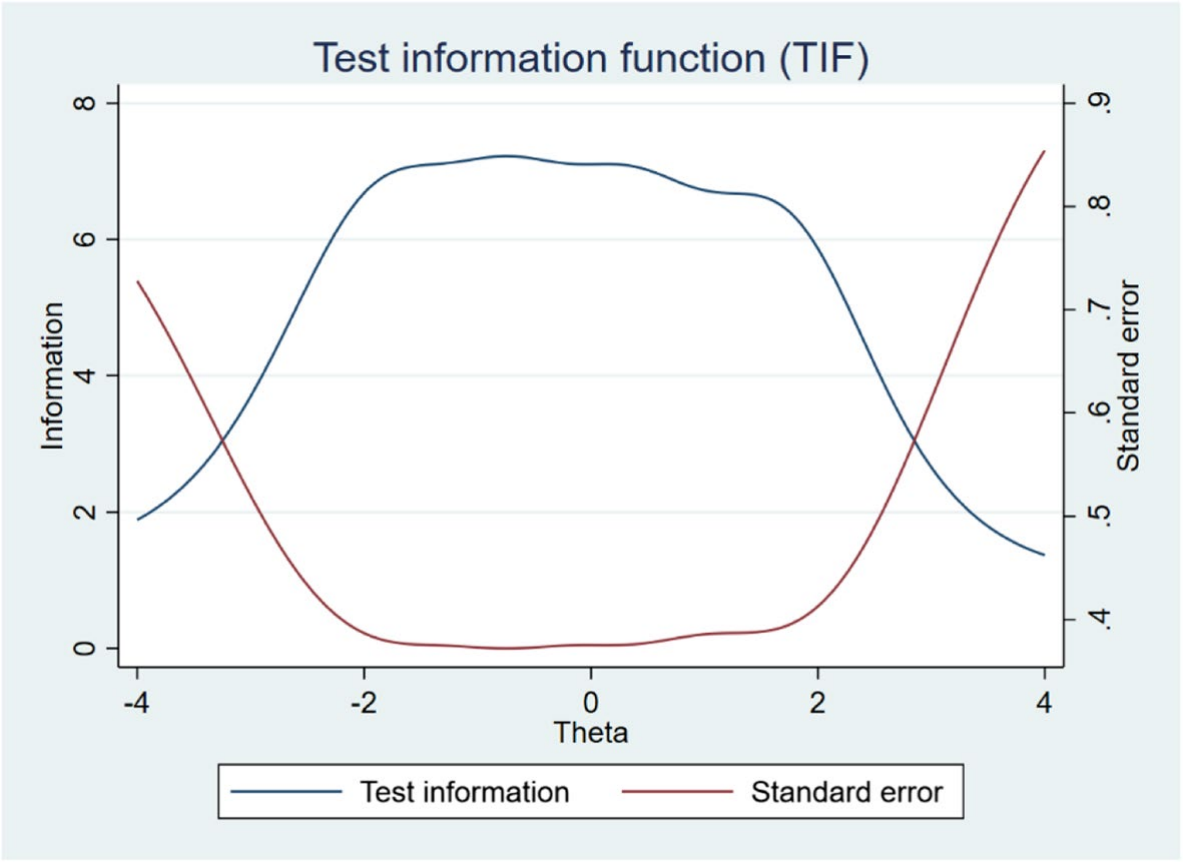
Test information function (TIF) and Standard error for SWEMWBS

The final step of the IRT analysis was to assess the overall performance of the scale. Overall, the scale performs well across the entire latent trait continuum, particularly in the key range from –2 to 2, exhibiting minimal measurement error (see Fig. 4). Figure 4 also clearly demonstrates that the scale items function cohesively and can effectively measure respondents with varying levels of mental well-being.

### Reliability and validity testing

The reliability of the SWEMWBS was tested using Cronbach’s alpha (*α*) and McDonald’s omega (*ω*). The results indicated good internal consistency of the scale, with *α* = 0.820 and *ω* = 0.824. Confirmatory factor analysis (CFA) with MLR estimation method was used to test construct validity. The results showed a good fit of the one-factor model to the data (RMSEA = 0.070, CFI = 0.957, SRMR = 0.029, χ^2^ (21) = 36070.211, *p* < 0.001) when the values of all parameters met the required criteria [30] (for more detail, see Figure A2 in the Appendix). The convergent validity was tested by the average variance extracted (AVE) and composite reliability coefficient (CR) [31] to determine the internal consistency of indicators measuring the same construct [32]. To achieve convergent validity, it is recommended that the AVE should be more than 0.5 and the CR should exceed 0.7 [33]. However, strict adherence to these values is not required. Convergent construct validity is considered sufficient as long as the AVE is less than 0.5 and the CR is greater than 0.6 [31]. This situation applies exactly to the results of this study, as the AVE was 0.402 but the CR was 0.821.

Finally, to assess the criterion-related validity of the SWEMWBS scale, correlations with other relevant measures were calculated (see Table 4). The results indicated a high level of criterion-related validity, as the correlations with all measures were large and statistically significant [34]. The largest positive correlations were found with the questions on happiness (0.60) and life satisfaction (0.59). In contrast, the PHQ-2 (− 0.42) and GAD-2 (− 0.49) measures of depression and anxiety showed negative correlations. These results are consistent with previous studies [e.g., 15, 35, 36].

**Table 4:** Correlations of the SWEMWBS with other relevant measures

	*r*	CI 95%
Life satisfaction	.59**	[.59; .60]
Happiness	.60**	[.59; .61]
General health	.39**	[.38; .40]
GAD-2	− .49**	[− .50; − .48]
PHQ-2	− .42**	[− .43; − .41]
BRS	.52**	[.51; .53]

Pearson correlation coefficient

^**^p < .01

## Discussion

The aim of this study was to validate and examine the psychometric properties of the widely-used SWEMWBS on a large scale representative sample of Czech adolescents. The main contribution of this study is threefold. First, there is still a lack of validation studies of mental well-being scales among adolescents that are considered vulnerable for mental health issues. Second, this is the first SWEMWBS validation study conducted in the central European region. Third, we combined CTT and IRT methods that represent two different but complementary measurement frameworks, an approach which provides a complex test of the scale.

All three assumptions of IRT (unidimensionality, local independence, and monotonicity) were met. Although residual correlations between several items were slightly higher (up to 0.28) in a few cases, the assumption of local independence is satisfied due to the positive wording and quality of the data. Using GRM (indicated better fit than GPCM), we found that the values of the discrimination parameter range from “moderate” to “very high” discriminative power. This is related to the information contribution by each item to the scale (see Fig. 1), as the most discriminative items, 2 and 4, can also be described as the most informative, while item 7 is the least informative. Threshold parameter values showed that the scale performs very well along the latent trait continuum, except for item 7, which covers more of the left (negative) side of the latent trait (mental well-being) continuum. The findings are consistent with those of a previous study conducted in the UK on adolescents [15]. Based on the analysis of the CCCs, we found that the response scale functions very well and has an adequate number of response categories. With the exception of item 7, all items had a clear peak and were not superimposed by the curve of another response category. Although item 7 did not function optimally (being the least informative and least discriminating item) its removal did not improve the quality of the scale. Further, the results revealed that the differences between categories were consistently minimal indicating that the response scale functioned effectively for all items.

The results of reliability tested by Cronbach’s alpha (*α*) and McDonald’s omega (*ω*) indicated good internal consistency of the scale. Additionally, CFA with MLR estimation method showed a good fit of the model to the data. The lowest factor loadings were found for item 7, which is consistent with the results of the study conducted on Swedish adolescents [37]. Despite the lower values of AVE (0.402), the CR values (0.821) were sufficient which suggest convergent validity of the scale. In line with previous studies [e.g., 15, 36], we found large correlations between SWEMWBS and happiness (0.60), life satisfaction (0.59), depression (− 0.42) and anxiety (− 0.49) suggesting that SWEMWBS can be considered a valid measure of mental health.

The mean score of mental well-being among Czech adolescents is 21.44, which is below the mean score observed among adolescents in other countries. For instance, adolescents in the UK exhibited a mean score of 23.57 for females and 23.17 for males [38]. Similarly, adolescents in Norway demonstrated a mean score of 24.90 [39]. Furthermore, adolescents in Ireland exhibited a mean score of 25.43, while those in Scotland a mean score of 24.55 [40]. Finally, study by Koushede et al. [35] found that adolescents in Denmark had a mean score of 25.80, and in Iceland a mean score of 23.60. Nevertheless, it is important to note that the age groups being compared are not identical, and that the above studies were conducted prior to the onset of the COVID-19 pandemic.

The results of our study also indicated that there were statistically significant differences in levels of mental well-being by gender. Men exhibited higher levels of mental well-being than women (22.67 vs. 20.28). Conversely, no differences were found based on the type of secondary school the students attended, as the mean mental well-being scores were 21.87 for gymnasiums, 21.41 for secondary technical schools, and 21.17 for secondary vocational schools. In a subsequent study, we intend to build upon these results and test the invariance of this scale across different groups (e.g., based on gender, type of study, ethnicity), as this type of analysis is currently uncommon.

## Conclusion

Following on from previous studies that have found SWEMWBS to be a reliable and valid instrument for measuring mental well-being, this study has shown that the Czech version also indicates good quality at a scale-level as well as at an item-level. In sum, the Czech version of the SWEMWBS was easy to administer and, based on the results of this study, represents a valuable tool for measuring mental well-being among Czech adolescents.

## Supplementary Information

Supplementary Material 1.

## Data Availability

Survey data are part of an ongoing project and are not publically available, yet. The data can be made available upon reasonable request. Kudrnáč, A., Hanzlová, R., Spitzerová, M., Aslan, K., & Bocskor, Á. (2024). Czech Education Panel Survey 1. Wave – student questionnaire [dataset] 10.14473/CSDA/OM42GN.

## Abbreviations

AIC: Akaike information criterion
AVE: Average variance extracted
BIC: Bayesian information criterion
BRS: Brief resilience scale
CASI: Computer Assisted Self Interviewing
CCC: Category characteristic curve
CFA: Confirmatory factor analysis
CI: Confidence interval
CR: Composite reliability coefficient
CTT: Classical Test Theory
CZEPS: Czech Education Panel Study
GPCM: General Partial Credit Model
GAD: General Anxiety Disorder
GRM: Graded Response Model
ICC: Item characteristic curve
IIF: Item information function
IRT: Item Response Theory
M: Mean
MLR: Maximum likelihood parameter estimates with standard errors
PCA: Principal component analysis
PHQ: Patient Health Questionnaire
SD: Standard deviation
SWEMWBS: Short Warwick‑Edinburgh Mental Well-Being Scale
TIF: Test information function
TRAPD: Translation, Review, Adjudication, Pretesting, Documentation
WEMWBS: Warwick‑Edinburgh Mental Well-Being Scale
WHO: World Health Organisation

## Footnotes

1 SWEMWBS is free to use, but protected by copyright. For commercial and non-commercial use, you must apply for a licence from University of Warwick at this website: https://warwick.ac.uk/fac/sci/med/research/platform/wemwbs/using. This study was registered on 1 June 2023.
