# Supplementary material for: Validation and psychometric evaluation of the Short Warwick-Edinburgh Mental Well-Being Scale (SWEMWBS) among Czech adolescents using Item Response Theory
Source: Health Qual Life Outcomes. 2024 Aug 19;22:66. doi: 10.1186/s12955-024-02280-9 (PMC11331616; doi:10.1186/s12955-024-02280-9)
Supplement: Supplementary file 1 — Supplementary Material 1. [file 12955_2024_2280_MOESM1_ESM.docx]

**APPENDIX**

**Table A1** Characteristics of research sample (*N* = 22,498)

|  | Frequency | Percent |
| --- | --- | --- |
| Gender |  |  |
| Male | 10,777 | 49.0 |
| Female | 11,047 | 50.2 |
| Other | 173 | 0.8 |
| Type of secondary school |  |  |
| Gymnasium | 4,180 | 18.6 |
| Secondary technical school | 13,138 | 58.4 |
| Secondary vocational school | 5,180 | 23.0 |
| Region (NUTS2) |  |  |
| Prague | 2,112 | 9.4 |
| Central Bohemia | 1,965 | 8.7 |
| Southwest | 2,120 | 9.4 |
| Northwest | 3,520 | 15.6 |
| Northeast | 3,079 | 13.7 |
| Southeast | 3,570 | 15.9 |
| Central Moravia | 3,697 | 16.4 |
| Moravian-Silesian | 2,435 | 10.9 |
| Parental education attainment |  |  |
| Primary or secondary education without GCSE | 4,009 | 19.4 |
| One highest secondary education without GCSE | 5,060 | 24.5 |
| Both secondary education with GCSE | 3,778 | 18.3 |
| One university degree | 4,868 | 23.6 |
| Both university degree | 2,935 | 14.2 |
| Subjective assessment of household income |  |  |
| Low income | 3,597 | 16.0 |
| Average income | 14,293 | 63.5 |
| High income | 2,705 | 12.0 |
| Don't know | 1,903 | 8.5 |

**Table A2** Results of principal component analysis (PCA): total variance explained

| Component | Initial Eigenvalues | | | Extraction Sums of Squared Loadings | | |
| --- | --- | --- | --- | --- | --- | --- |
|  | Total | % of Variance | Cumulative % | Total | % of Variance | Cumulative % |
| 1 | 3.389 | 48.414 | 48.414 | 3.389 | 48.414 | 48.414 |
| 2 | .831 | 11.875 | 60.289 |  |  |  |
| 3 | .725 | 10.363 | 70.651 |  |  |  |
| 4 | .656 | 9.367 | 80.018 |  |  |  |
| 5 | .531 | 7.584 | 87.602 |  |  |  |
| 6 | .449 | 6.410 | 94.012 |  |  |  |
| 7 | .419 | 5.988 | 100,000 |  |  |  |
| Extraction Method: Principal Component Analysis. | | | | | | |

**Figure A1** Scree plot from PCA


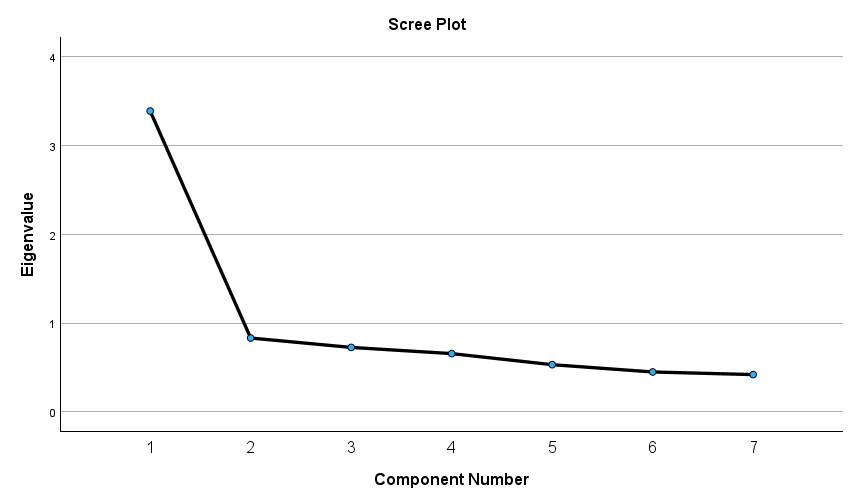


**Table A3** Residual correlation between pairs of SWEMWBS items

|  | item 1 | item 2 | item 3 | item 4 | item 5 | item 6 | item 7 |
| --- | --- | --- | --- | --- | --- | --- | --- |
| item 1 | 1.00 | –.02 | –.21 | –.22 | –.18 | –.08 | –.04 |
| item 2 |  | 1.00 | –.18 | –.28 | –.28 | –.08 | –.13 |
| item 3 |  |  | 1.00 | –.10 | –.24 | –.10 | –.16 |
| item 4 |  |  |  | 1.00 | –.07 | –.23 | –.14 |
| item 5 |  |  |  |  | 1.00 | –.13 | .05 |
| item 6 |  |  |  |  |  | 1.00 | .00 |
| item 7 |  |  |  |  |  |  | 1.00 |

**Table A4** Comparison of model fit: GPCM vs. GRM

|  | Log-likelihood | AIC | BIC |
| --- | --- | --- | --- |
| GPCM | –201022.4 | 402114.9 | 402395.6 |
| GRM | –200146.2 | 400362.5 | 400643.2 |

*GPCM* General Partial Credit Model, *GRM* Graded Response Model, *AIC* Akaike information criterion, *BIC* Bayesian information criterion

**Table A5** Differences in *b*-parameters between items

|  | b1/b2 | b2/b3 | b3/b4 | b1/b2 – b2/b3 | b2/b3 – b3/b4 |
| --- | --- | --- | --- | --- | --- |
| item 1 | 1.13 | 1.44 | 1.48 | 0.31 | 0.04 |
| item 2 | 1.09 | 1.23 | 1.34 | 0.14 | 0.11 |
| item 3 | 1.28 | 1.09 | 1.42 | 0.19 | 0.33 |
| item 4 | 1.07 | 0.92 | 1.17 | 0.15 | 0.25 |
| item 5 | 1.21 | 1.19 | 1.37 | 0.02 | 0.18 |
| item 6 | 1.45 | 1.50 | 1.82 | 0.05 | 0.32 |
| item 7 | 1.55 | 1.73 | 1.86 | 0.18 | 0.13 |

**Figure A2** Results of confirmatory factor analysis (CFA) – standardised estimates

Residual variances


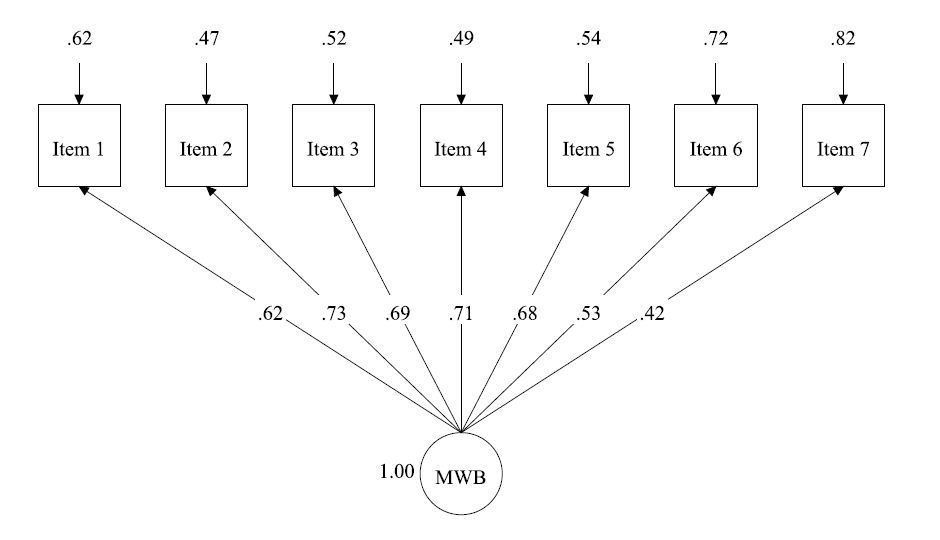


Factor loadings

*Note*.  The values between the measured concept (MWB) and the items are factor loadings. The values for each item indicate the residual variance.
